# Modulation of atrazine-induced chromosomal aberrations and cyclin-dependent kinases by aqueous extract of *Roylea cinerea* (D.Don) Baillon leaves in *Allium cepa*

**DOI:** 10.1038/s41598-022-16813-z

**Published:** 2022-07-22

**Authors:** Farhana Rashid, Davinder Singh, Shivani Attri, Prabhjot Kaur, Harneetpal Kaur, Pallvi Mohana, Jahangeer Quadar, Adarsh Pal Vig, Astha Bhatia, Balbir Singh, Harpreet Walia, Saroj Arora

**Affiliations:** 1 Department of Botanical and Environmental Sciences, Guru Nanak Dev University, Amritsar, Punjab 143005 India; 2 Department of Pharmaceutical Sciences, Guru Nanak Dev University, Amritsar, Punjab India; 3 Department of Botany and Environment Studies, DAV University, Jalandhar, Punjab India

**Keywords:** Environmental impact, Mitosis

## Abstract

*Roylea cinerea* (D.Don) Baillon an indigenous medicinal plant of *Lamiaceae* family used for the treatment of several diseases. In the present study, its aqueous (leaves) extract was tested for genoprotective action against atrazine-induced chromosomal aberrations in the root tip cells of *Allium cepa*. Atrazine is a herbicide of triazine class commonly used to inhibit the growth of broad leaf and grassy weeds. In order to find the concentration of atrazine that exhibits maximum toxicity, its different concentrations (1, 5 and 10 µg/mL) were tested. It was observed that 10 µg/mL concentration was more toxic as it reduced the mitotic index and also increased the chromosomal aberrations. Among all the tested concentrations of aqueous (leaves) extracts (0.25. 0.5, 1.0, 1.5 and 3.0 µg/mL), the3.0 µg/mL concentration in both modes of experiments i.e. pre and post showed a significant reduction in chromosomal aberrations induced by atrazine. To understand the mechanism of protection by plant extract on atrazine-induced chromosomal abnormalities the RT-qPCR studies were conducted to observe the expression of marker genes Cyclin-dependent kinases (CDKs) (CDKA:1, CDKB2:1 and CDKD1:1. For this, the RNA was extracted from root tips treated with extract along with atrazine by TRIzol^®^. It was observed that aqueous extract of *Roylea cinerea* (D.Don) Baillon leaves upregulated the CDKs gene expression in both the modes i.e. pre and post treatments. A critical analysis of results indicated that aqueous extract ameliorated the chromosomal aberrations caused by atrazine which may be be due to the increased expression level of CDKs genes.

## Introduction

Atrazine (2-chloro-4-ethylamino-6-isopropylaminotriazine) is a triazine class herbicide which is commonly used to eradicate the weeds growing among the several crops like sorghum, pineapple, maize, and sugarcane, as well as in conifer reforestation plantings^1^. Furthermore, it is one of the most prevalent contaminants found in both ground and surface waterways and agricultural streams and rivers^2–5^.

Atrazine has been linked to genotoxicity and ecotoxicology in several studies^6,7^. In the *Allium cepa* test, Chinese hamster ovary cells, and human lymphocytes, atrazine is clastogenic and is suspected of causing cancer in human workers^6,8,9^. Atrazine is harmful to a variety of amphibian species and some studies suggest that it is an endocrine disrupter^10–12^. As atrazine is widely used in India and is known to be one of the major water pollutants, therefore, determining its genotoxic potential is critical. Excessive agrochemical active ingredient accumulation harms plant health and causes oxidative and genotoxic damage to essential biomolecules in plant cells due to the production of reactive oxygen species (ROS)^13,14^. An increase in ROS in plant cells triggers both oxidative and genotoxic stress responses resulting in cytotoxicity and damage to various cellular components such as proteins, membranes, and nucleic acids, as well as cell growth and development suppression^15–18^.

Higher plants possess secondary metabolites that have been proven to provide a nearly limitless supply of compounds with biological action^19^. Several plants and their products have been screened by researchers for their antimutagenic and antigenotoxic properties. Antimutagenic agents are natural compounds that are used to minimize the genotoxic effects of mutagenic or carcinogenic chemicals^20^.

It has also been hypothesized that using antimutagens and anticarcinogens in everyday life is an effective method of preventing human cancer and genetic illnesses^21^. Medicinal plants contain bioactive substances that can slow or stop the progression of cancer in its early stages^22^. Higher plants, which have long been employed in traditional medicines, are now being studied for their ability to modulate the activity of ambient genotoxicants^23^.

Keeping in view the medicinal importance of plants, *Roylea cinerea* (D.Don) Baillon was selected for the present study. *Roylea cinerea* (D.Don) Baillon plant of the Lamiaceae family, is an Indian native herb that grows at altitudes of 1200–3700 m in the Western Himalayas and Nepal's foothills. This plant has been used to treat diabetes, malaria, and skin problems, as well as a febrifuge and tonic for contusions^24,25^. The antiplasmodial action of petroleum ether and chloroform extracts from *Roylea cinerea* (D.Don) Baillon leaves has been documented^26^. Several phytochemical compounds like labdane diterpenoids: calyenone, epicalyone, calyone, and precalyone, cinereanoid A, cinereanoid B, moronic acid, cinereanoid C, cinereanoid D, b-lactam, flavonoid glycosides: rutin, isoquercetin, nicotiflorin, martynoside, undatuside A and 50-b-d-glucopyranosyloxyjasmonic acid, from chloroform fraction: pilloin, 1-methylindole-3-carboxaldehyde, b-sitosterol, and stigmasterol have been isolated from the aerial parts of the plants^27–30^.

In a study conducted on mice with P-388 lymphocytic leukemia, the compound precalyone (a diterpene) isolated from *Roylea cinerea* demonstrated anticancer activity up to 143 percent at a dose of 50 mg/kg^31,32^. Furthermore, a target-oriented binding analysis to active binding sites of Hsp90 and Hsp70 proteins was described in another work, which revealed probable dual binding affinity of cinereanoid D at 0.1 mg/mL and 1 mg/mL concentrations to both proteins^30^. Till date, no study has been done on the anti-genotoxic potential of aqueous extract of leaves of *Roylea cinerea* (D.Don) Baillon against the atrazine-induced chromosomal aberrations on the *Allium cepa* root tip assay. For this purpose, the short-term *Allium cepa* chromosomal aberration assay was used. Although, *Allium cepa* has been used by many researchers as a plant model system to evaluate the effects of many substances, the studies demonstrating the molecular basis underlying these effects are still lacking and there is a need to study the effect of atrazine at the gene level. Keeping this in view, the present study was planned to evaluate the antigenotoxic potential of aqueous extract of *Roylea cinerea* (D.Don) Baillon leaves against atrazine-induced toxicity in root tip cells of *Allium cepa.*

Despite indications of ethnomedicinal and traditional usage, this plant has only undergone a cursory scientific examination. The utilization of traditional knowledge for the benefit of a greater population will be aided by well-planned studies to prove the scientific basis for using the plant to mitigate the damaging effects of pesticides.

## Materials and methods

### Chemicals

Herbicide Atrazine (C_8_H_14_ClN_5_) was purchased from Sigma-Aldrich (St. Louis, MO, USA) (Cas no: 1912-24-9) (Table 1).

**Table 1:** Some chemical properties of atrazine.

Chemical name	Chemical formulae	(IUPAC name)	Chemical structure	Molecular weight	Used as
Atrazine	C_8_H_14_ClN_5_	2-Chloro-4-ethylamino-6-isopropylaminotriazine		215.68 g/mol	Herbicide

### Collection of plant material

The plant used in the study i.e.* Roylea cinerea* (D.Don) Baillon is a commonly growing weed which was collected from District Palampur, Himachal Pradesh, India. There are no reports regarding its inclusion in the list of endangered and extinct species [Website: http://www.bsienvis.nic.in/Database/RedlistedPlants_3940.aspx (ENVIS Resource Partner on Biodiversity hosted by Botanical Survey of India, Kolkata, West Bengal and sponsored by Ministry of Environment, Forest and Climate Change, Govt of India)]. Also, non-lethal and responsible collection guidelines were adopted during the collection of the study material *Roylea cinerea* (D.Don) Baillon leaves and submitted as a voucher specimen (Accession no. 7376) in Herbarium, Department of Botanical and Environmental Sciences, Guru Nanak Dev University, Amritsar, Punjab. The identification was done by Dr. Amarjit Singh Soodan (Taxonomist) and Mr. Ram Prasad (Senior lab technician), of the Department of Botanical and Environmental Sciences, Guru Nanak Dev University, Amritsar, Punjab^33^.

### Preparation of the aqueous extract

*Roylea cinerea* (D.Don) Baillon leaves were collected and thoroughly cleaned under tap water. The leaves were then kept at room temperature for a few days until they get completely dried. The completely air-dried leaves were coarsely powdered and their maceration was done in methanol for 2–3 days with stirring at regular intervals. Into the crude methanolic extract distilled water and an equal amount of hexane were added and the whole mixture was shaken vigorously. The mixture was then kept undisturbed and allowed to separate into layers. The hexane fraction was obtained by collecting and concentrating the upper hexane layer in a rotary evaporator. The remaining aqueous layer was subjected to fractionations by a series of solvents based on polarity viz., Diethyl-ether < Ethyl acetate < *n*-butanol < water^33^. The final fractionated aqueous extract was used in the present study.

### Analysis of aqueous extract by high-performance liquid chromatography (HPLC) for the presence of phytochemicals

High-performance liquid chromatography (HPLC) analysis was carried out on a sample of the aqueous (leaves) extracts of *Roylea cinerea* (D.Don) Baillon at the Emerging Life Sciences Department). HPLC was performed with the HPLC system (Shimadzu, Kyoto, Japan), consisting of a quaternary pump with a vacuum degasser, stable thermostatic column compartment, auto-sampler, and a UV detector. Reverse phase chromatographic analysis was carried out under gradient conditions using a C18G column (4.6 mm × 250 mm) packed with 5 μm diameter particles at 30 °C temperature. The mobile phase contained binary solvent A: Water and Solvent B: Acetonitrile. The Flow rate was maintained at 1.0 mL/min with an injection volume of 20 μL and peaks were detected at 280 nm. Gallic acid, catechin, rutin, quercetin, ellagic acid, umbelliferone and kaempferol were used as standards. All the solutions were sonicated, and filtered through a 0.22 μm filter membrane before HPLC analysis. Test samples (plant extracts) were prepared in HPLC grade methanol (10 mg/mL) in a volumetric flask and further sonicated to dissolve completely. After complete dissolution, samples were filtered through a 0.22 μm filter and filled in HPLC vials for analysis.

### Antigenotoxic potential of *Roylea cinerea* (D.Don) Baillon leaves

In the present investigation, aqueous leaves extract of *Roylea cinerea* (D.Don) Baillon was analyzed for its protective activity against the genotoxic effect of atrazine. The concentration at which the atrazine exhibited maximum chromosomal aberrations was selected. For this, onion bulbs in triplicate were first grown in tap water for 48 h and then the roots were treated with different concentrations of atrazine (1, 5 and 10 µg/mL) for the next 24 h. After treatment, the roots were thoroughly washed and checked for their length, number, mitotic index, and chromosomal aberrations. For root length measurement, 10 healthy roots from three onions at each concentration were cut and root length was measured (cm) and used as an index of general toxicity (root growth inhibition). From the weighted average for each concentration and the control, the percentage root growth inhibition with the negative control was calculated and the EC_50_ value was determined^34^. The effective concentration among the three used were 10 µg/mL as it showed reduced root growth.

In another experiment, the antigenotoxic potential of aqueous extract of leaves of *Roylea cinerea* (D.Don) Baillon alone was also determined. For this, the healthy onion bulbs in triplicate were first grown in tap water for 48 h and after 48 h bulbs with roots were treated with different concentrations of aqueous extract (0.25, 0.5, 1.0, 1.5, and 3.0 µg/mL) for next 24 h. After treatment, roots were thoroughly washed and checked for their length, number, mitotic index, and chromosomal aberrations.

The genoprotective activity of *Roylea cinerea* (D.Don) Baillon, against 10 µg/mL concentration of atrazine was also tested. Two types of treatments i.e. pre-treatment and post-treatment were given. In pre-treatment, the roots were first treated with different concentrations of extract (0.25, 0.5, 1.0, 1.5, and 3 µg/mL) for 48 h, and then the same roots after washing were treated with atrazine10 µg/mL for another 24 h. In post-treatment, the root tips were first treated with atrazine 10 µg/mL for 48 h and after washing they were treated with different concentrations (0.25, 0.5, 1.0, 1.5, and 3.0 µg/mL) of extract for 24 h. After the treatment, the rootlets were cut, washed, and immediately fixed in glacial acetic acid: ethanol (1:3) fixative and kept for 24 h. After 24 h, the rootlets were taken out and were transferred to 70% ethanol and stored at 4 °C until use. The treatment of root tips with tap water alone served as negative control and root tips treated with sodium azide i.e. (5 µg/mL) served as a positive control. For cytological studies, the root tips were hydrolyzed in 1 N HCl (5 min) and then washed and stained with 2% acetocarmine (1:9) and warm gently^35^. All slides were prepared by gently squashing two to three root tips on each slide and then viewed under a compound light microscope.

The percentage of the mitotic index, as well as the frequencies of different mitotic abnormalities, were determined. Mitotic index was expressed in percentage by counting 1000 cells per slide in triplicate (for atrazine and sodium azide) and150 cells per slide for aqueous extract respectively. Both physiological aberrations (PA) and clastogenic aberrations (CA) were recorded as; c-mitosis, stickiness, delayed anaphase/telophase, vagrants, laggards, chromosome fragments, chromosome bridges, and chromosome breakage.

The mitotic index was calculated as:

$$ {\text{M}}.{\text{I}}. \, = \frac{{\text{Total number of dividing cells}}}{{\text{Total number of counted cells}}} \times 100 $$

Chromosomal aberration frequency was calculated as:

$$ {\text{Aberration frequency}} = \frac{{\text{Total number of aberrant cells}}}{{\text{Total number of dividing cells}}} \times 100 $$

### Statistical analysis

All experiments were repeated thrice and pooled data were tabulated as average ± SD. The statistical analyses were performed using the (SPSS 16.0) software. Data on root length, root number, mitotic index, and chromosomal aberrations were compared using analysis of variance (ANOVA) followed by the Turkey test (p < 0.05).

### DNA damage analysis with real time-quantitative polymerase chain reaction (RT-qPCR)

#### RNA isolation and quantitative real-time polymerase chain reaction

Total RNA was extracted from the control and treated roots with TRIzol^®^ reagent and liquid nitrogen method with some modifications^36^. The purity and concentration of RNA were determined using a Nanodrop 2000 (Thermo scientific). An equal concentration of RNA was used to synthesize cDNA by using the protocol of LUNA Universal one-step Reverse Transcriptase Polymerase Chain Reaction (RT-PCR Kit**).** Briefly, the master mixture consisted of dye (reaction mixture), enzyme mixture, water, and template. Then again quantification of cDNA was done on Nanodrop 2000 (Thermo scientific).

#### Real time-quantitative polymerase chain reaction (RT-qPCR)

Normal PCR was used to amplify the number of copies of specific cDNA sequences. All primers used for (RT– qPCR) are listed in Table 2. They were designed based on sequencing data of expressed sequence tags (ESTs) from *Allium cepa* L. (2n = 16) database of selected genes on the website of the National Center for Biotechnology Information (NCBI). Primers were designed using the Primer 5 software following the manufacturer’s guidelines for primer design. Primers were purchased from Bioserve (A REPROCELL COMPANY). Samples of cDNA were standardized on the housekeeping gene viz. (TUBULIN β) with run ID (566464). (TUBULIN β) run ID (566464) was used as an internal constitutively expressed control (reference gene) using a gene-specific primer in PCR. For a typical PCR reaction, 20 ng cDNA was used as a template in 9 μL reaction volume according to instructions supported with MyTaqTM Red Mix 2 × (BIOLINE). Using Luna One-Step RT-qPCR (New England BioLabs), the gene expression of CDKs (CDKA:1, CDKB2:1, and CDKD1:1) was recorded. The results were expressed in comparison to the expression levels of an internal reference gene tubulin in each sample using the 2^−ΔΔCt^ method^37^.

**Table 2:** Genes studied and tubulin as housekeeping gene: (names, Run ID (NCBI) and primers sequences).

Sr. No	Gene	Run ID	Primer sequence 5′–3′	Length
1	CDKA:1 F	566458	CGGGTAAATAGGTAACAA	18
2	CDKA;1 R	566459	CTGAGGTGTCTTATTAGT	18
3	CDKB2;1 F	566460	AGCATTCGCAAAATGGAGAT	20
4	CDKB2;1 R	566461	TTTAGAGAGCGATGGACGAGG	21
5	CDKD1;1 F	566462	GCTCCCAAGACCAGTTTCCA	20
6	CDKD1;1 R	566463	CCAGACTTTCCTCGGTCAGG	21
7	TUBULIN β-2 F	566464	ACACCAGACATAGTAGCAGAAATCAAG	27
8	TUBULIN β-2 R	566465	GAGCCTTACAACAACGCTACTCTGTCTGTC	30

### Compliance with ethical standards

This manuscript is original, has not been published before and is not currently being considered for publication elsewhere. Accepted principles of ethical and professional conduct have been followed while executing this research work. No experiment was carried out on humans or animals to accomplish this research work.

### Consent to participate

No human participants were required/used to carry out the reported research work. As there are no participants, consent to participate is not required.

### Consent to publish

We the undersigned declare that this manuscript is original, has not been published before, and is not currently being considered for publication elsewhere. We confirm that the manuscript has been read and approved by all named authors and that there are no other persons who satisfied the criteria for authorship but are not listed. We further confirm that the order of authors listed in the manuscript has been approved by all of us. We understand that the Corresponding Author is the sole contact for the Editorial process. He/she is responsible for communicating with the other authors about progress, submissions of revisions, and final approval of proofs.

## Results

**Figure 1:**
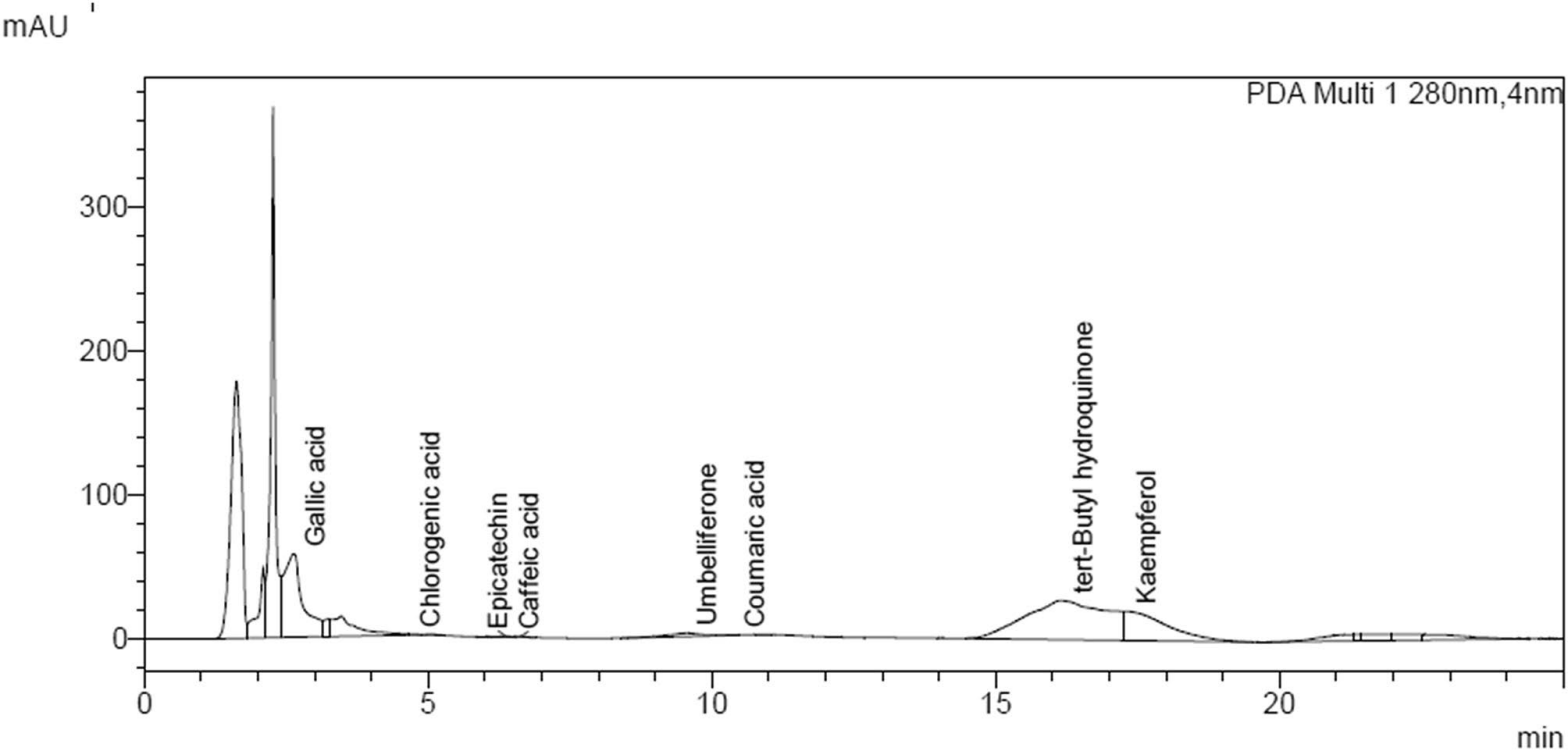
Representative profile of HPLC chromatogram of different polyphenols detected in aqueous extract of leaves of *Royale cinerea* (D.Don) Baillon, where (peak 1): Gallic acid , (peak 2): Chlorogenic acid, (peak 3): Epicatechin, (peak 4): Caffeic acid, (peak 5): Umbelliferone, (peak 6): Coumaric acid, (peak 7): tert-Butyl hydroquinone (peak 8), Kaempferol respectively were recorded at 280 nm.

The aqueous extract of leaves of *Roylea cinerea* was prepared and tested for their potential as a modulatory agent against atrazine-induced abnormal expression of Cyclin-dependent kinases in the root tip cells of *Allium cepa*. In addition, the root tips treated with aqueous extract alone, atrazine, and in combination (Pre and Post) with extracts were assessed for its effect on different parameters viz. root growth, mitotic index, and Chromosomal aberrations. The preliminary phytochemical analysis showed the presence of eight polyphenolic compounds viz; Gallic acid, Chlorogenic acid Epicatechin, Caffeic acid, Umbelliferone, Coumaric acid, tert-Butyl hydroquinone, and Kaempferol (Fig. 1). The concentration of different polyphenols were; Gallic acid: 95.775 mg/L; Chlorogenic acid: 0.481 mg/L; Epicatechin: 1.433 mg/L; Caffeic acid: 0.314; Umbelliferone: 22.294 mg/L; Coumaric acid: 0.201 mg/L; tert-Butyl hydroquinone: 437.784 mg/L and Kaempferol: 449.027 mg/L (Table S1). Identification of these compounds was done by comparing their retention time and UV absorption spectrum with those of the commercial standards run along with extract.

**Figure 2:**
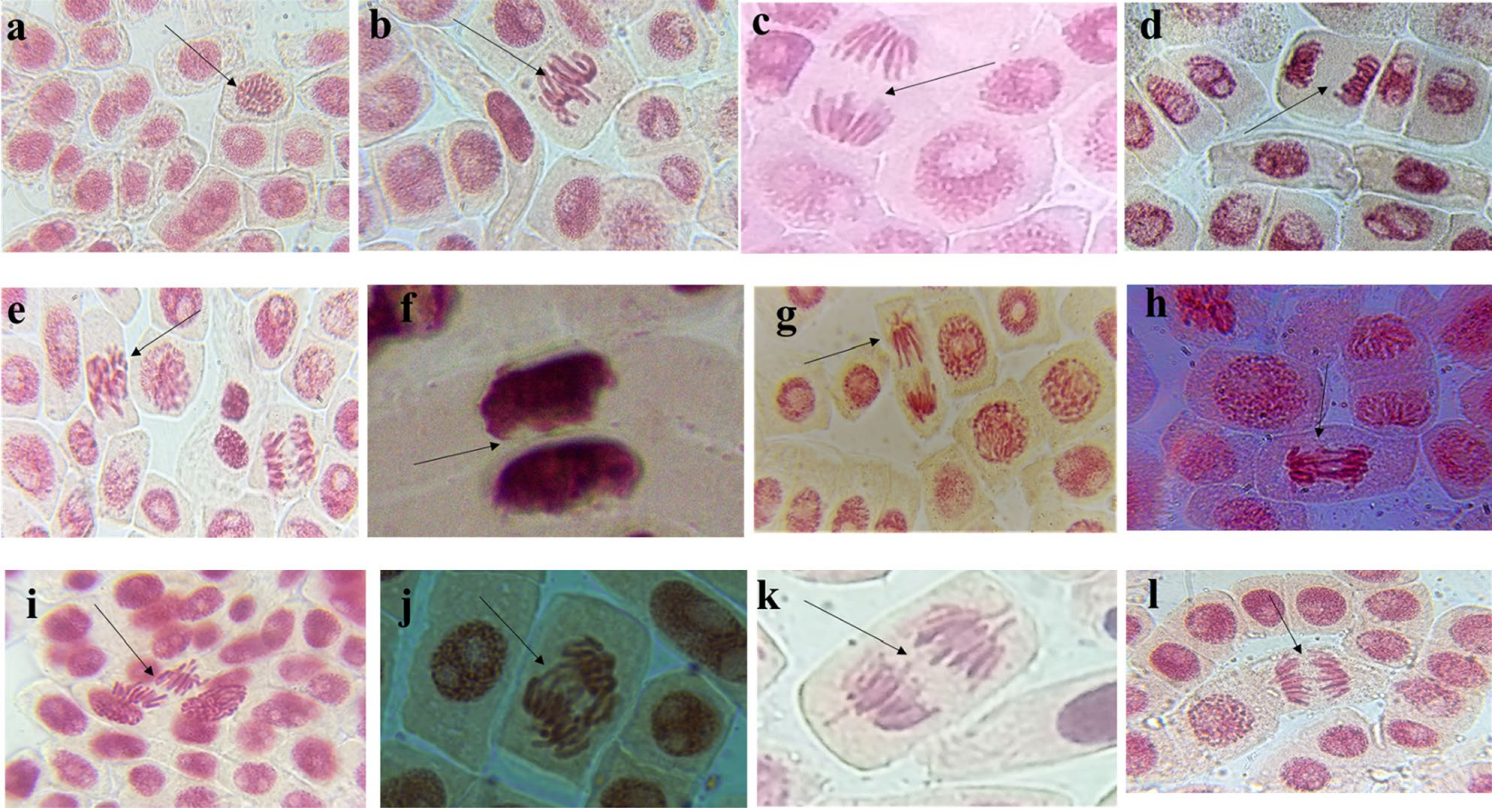
Normal stages of mitosis. (**a**) prophase; (**b**) metaphase; (**c**) anaphase; (**d**) telophase; chromosomal aberrations; (**e**) c-metaphase; (**f**) stickiness at telophase; (**g**,**k**) vagrant chromosomes; (**h**) chromatin bridge; (**i**) laggard chromosomes; (**j**) chromosomes fragments/breaks; (**l**) delayed anaphase with bridge.

In the present study, significant dose-dependent growth retardation of *Allium cepa* roots due to atrazine treatment was observed. Data on the root length and root number is presented in Table 3. A maximum inhibition in terms of root number and root length as well as root growth occurred at 10 µg/mL of concentration of atrazine. The maximum decrease in average root length (2.69 cm) was recorded at 10 µg/mL concentration of atrazine. Furthermore, there was a significant reduction in the mitotic index values on the treatment of root tips with atrazine. A maximum reduction in percentage of the mitotic index was also observed at 10 µg/mL concentration of atrazine. The highest decrease in the percentage of mitotic index value (30.50%) was noted at 10 µg/mL concentration of atrazine in comparison to (57.10%) for control (Table 4). A significant difference in percentage frequency of chromosomal aberrations was also observed. Among different chromosomal aberrations, the percentage frequency of physiological chromosomal aberrations (15.13%, 20.73 and 46.33%) was found to be higher in comparison to clastogenic chromosomal aberrations (13.94%, 23.76%, and 24.69%) at 1, 5 and 10 µg/mL concentrations of atrazine respectively in treatment groups (Table 5). Chromosomal aberrations were also seen at different mitotic stages in the *Allium cepa* cells in all the tested concentrations (1, 5 and 10 µg/mL) of atrazine. The most frequent types of aberrations observed in cells treated with concentrations (1, 5, and 10 µg/mL) of atrazine included stickiness, c-mitosis, vagrant chromosomes, laggards, delayed anaphase, chromosomal breaks, chromosomal bridges, chromosomal fragments (Fig. 2). In the present study, as the reduction in root growth and mitotic index, as well as an increase in chromosomal aberrations, occurred at 10 µg/mL concentration of atrazine, therefore this concentration was selected to study the ameliorating potential of aqueous extract of leaves of *Roylea cinerea* (D.Don) Baillon in the root tips cells of *Allium cepa*. But before this, the antigenotoxic activity of aqueous extract of leaves of *Roylea cinerea* (D.Don) Baillon alone in the root tips cells of *Allium cepa* was also tested. The results of root length, root number, and mitotic index are given in Tables 3 and 6. It was found that the aqueous extract of leaves of *Roylea cinerea* (D.Don) Baillon alone has shown the maximum increase in the root number, root length, and mitotic index values at 0.25, 0.5, 1.0, 1.5, and 3.0 µg/mL of extract concentrations in comparison to atrazine and sodium azide. Moreover, the percentage frequency of chromosomal aberration was also observed to be minimum for 0.25, 0.5, 1.0, 1.5 and 3.0 µg/mL, tested concentrations of extract alone in comparison to atrazine (Table 6).

**Table 3:** Effects of atrazine, sodium azide and plant extract on *Allium cepa* root growth.

Treatment groups	Concentrations µg/mL	Average root number ± SD	Average root length (cm) ± SD	TRG (%) ± SD	Percentage inhibition of TRG ± SD
NC	Tap water	47.67 ± 1.17^a^	5.07 ± 0.07^g^	87.03 ± 10.34^g^	0.00 ± 0.00^a^
ATZ	1	26.67 ± 7.57^ab^	4.47 ± 0.53^defg^	79.44 ± 5.67^defg^	12.97 ± 10.34^abcd^
	5	27.00 ± 10.00^ab^	4.08 ± 0.29^bcdef^	52.33 ± 6.91^bcdef^	20.56 ± 5.67^bcdef^
	10	19.33 ± 10.07^a^	2.69 ± 0.36^a^	71.47 ± 1.57^a^	47.67 ± 6.91^g^
SA	1	28.00 ± 3.61a^b^	3.67 ± 0.08^bcde^	62.48 ± 3.40^bcde^	28.53 ± 1.57^cdef^
	5	18.33 ± 6.11^a^	3.21 ± 0.18^ab^	68.09 ± 15.12^ab^	37.52 ± 3.40^fg^
	10	23.33 ± 4.51^ab^	3.50 ± 0.78^abc^	69.07 ± 1.97^abc^	31.91 ± 15.12^efg^
PE	0.25	33.33 ± 6.11^abc^	3.55 ± 0.10^abcd^	74.97 ± 5.16^abcd^	30.93 ± 1.97^defg^
	0.5	35.00 ± 5.00^abc^	3.85 ± 0.27^bcde^	81.32 ± 2.88^bcde^	25.03 ± 5.16^cdef^
	1.0	34.67 ± 1.53^abc^	4.18 ± 0.15^cdefg^	88.13 ± 2.48^cdefg^	18.68 ± 2.88^bcde^
	1.5	39.0 ± 6.56^bc^	4.53 ± 0.13^efg^	95.27 ± 1.65^efg^	11.87 ± 2.49^abc^
	3.0	39.67 ± 7.57^bc^	4.90 ± 0.09^fg^	77.47 ± 14.41^fg^	4.73 ± 1.66^ab^

*NC* negative control, *ATZ* atrazine, *SA* sodium azide, *TRG* total root growth.

Data are mean ± SD of three replicates. Different alphabets (a, b, c, d, e, f and g) are significantly different at (p < 0.05), as determined by Tukey HSD test.

**Table 4:** Percentage of mitotic cell division in *Allium cepa* root tips after treatment with atrazine and sodium azide.

Treatment groups	Concentrations µg/mL	Total counted cells	Total diving cells	Normal cells	Mitotic index (%) ± SD	Total aberrant cells	(%) Frequency of aberrant cells ± SD
NC	Tap water	3000	1713	1700	57.10 ± 11.09^b^	13	0.79 ± 0.25^a^
ATZ	1	3000	1434	1017	47.83 ± 4.93^ab^	417	29.11 ± 2.41^ab^
	5	3000	955	530	31.83 ± 7.10^a^	425	44.83 ± 10.40^bc^
	10	3000	915	265	30.50 ± 2.27^a^	650	71.35 ± 6.56^c^
SA	1	3000	1023	565	34.10 ± 10.69^ab^	458	44.78 ± 16.44^bc^
	5	3000	1057	578	35.23 ± 4.43^a^	479	45.86 ± 7.74^bc^
	10	3000	1011	618	33.70 ± 9.90^a^	393	38.87 ± 18.65^b^

*NC* negative control, *ATZ* atrazine, *SA* sodium azide.

Data are mean ± SD of three replicates. Different alphabets (a, b and c) are significantly different at (p < 0.05), as determined by Tukey HSD test.

**Table 5:** Chromosomal aberrations observed in *Allium cepa* roots cells treated with different concentrations of atrazine and sodium azide.

Sample code	Conc µg/mL	TC	TDC	NC	Physiological aberrations (PA)							Clastogenic aberrations(CA)							MI
					Cm	St	Vg	Lg	Da	TPA		Cf	Cb	Ck	TCA		TPA + TCA		
										No.	%				No.	%	No.	%	
NC		3000	1713	1700	1	3	2	1	4	11	0.64	–	1	1	2	0.11	13	0.79 ± 0.25^a^	57.10 ± 11.09^b^
ATZ	1	3000	1434	1017	56	65	20	37	39	217	15.13	78	83	39	200	13.94	417	29.11 ± 2.41^ab^	47.83 ± 4.93^ab^
	5	3000	955	530	63	58	29	15	33	198	20.73	93	78	56	227	23.76	425	44.83 ± 10.40^bc^	31.83 ± 7.10^a^
	10	3000	915	265	119	104	49	68	84	424	46.33	85	69	72	226	24.69	650	71.35 ± 6.56^c^	30.50 ± 2.27^a^
SA	1	3000	1023	565	70	35	29	39	37	210	20.52	96	87	65	248	24.24	458	44.78 ± 16.44^bc^	34.10 ± 10.69^ab^
	5	3000	1057	578	83	52	28	38	53	254	24.03	78	81	66	225	21.28	479	45.86 ± 7.74^bc^	35.23 ± 4.43^a^
	10	3000	1011	618	55	67	32	21	37	212	20.96	69	65	47	181	17.90	393	38.87 ± 18.65^b^	33.70 ± 9.90^a^

*NC* negative control, *ATZ* atrazine, *SA* sodium azide, *TC* total cells, *TDC* total dividing cells, *NC* normal cells, *Cm* C-mitosis, *St* stickiness, *Vg* vagrant, *Lg* laggard, *Da* delayed anaphase, *Cf* chromosome fragments, *Cb* chromosome bridge, *Ck* chromosome break, *TPA* total physiological aberration, *TCA* total clastogenic aberrations, *MI* mitotic index. Data are mean ± SD of three replicates. Different alphabets (a, b and c) are significantly different at (p < 0.05), as determined by Tukey HSD test.

**Table 6:** Antigenotoxic potential of aqueous extract of leaves of *Royale cinerea* (D.Don) Baillon alone by *Allium cepa* root tip assay.

Sample code Conc. µg/mL		TC	TDC	NC	Physiological aberrations (PA)							Clastogenic aberrations (CA)							MI ± SD
					Cm	St	Vg	Lg	Da	TPA		Cf	Cb	Ck	TCA		TPA ± TCA		
										No.	%				No.	%	No.	%	
NC	Tap water	450	271	264	2	2	–	1		5	1.85	1	–	1	2	0.74	7	2.59 ± 0.56^a^	60.22 ± 7.19^a^
ATZ	10	450	143	54	24	16	7	4	5	56	39.16	11	17	5	33	23.08	89	66.01 ± 24.49^a^	31.78 ± 8.34^a^
PE	0.25	450	178	165	3	2	1	2	1	9	5.06	1	1	2	4	2.25	13	7.30 ± 3.26^a^	39.56 ± 9.57^ab^
	0.5	450	195	178	1	2	3	2	–	8	4.10	3	2	4	9	4.62	17	8.71 ± 3.04^a^	43.34 ± 14.05^ab^
	1.0	450	213	201	2	3	1	1	3	10	4.69	1	–	1	2	0.94	12	5.63 ± 2.91^a^	47.33 ± 10.26^ab^
	1.5	450	222	212	1	1	2	1	–	5	2.25	2	2	1	5	2.25	10	4.50 ± 2.54^a^	49.33 ± 4.37^ab^
	3.0	450	254	245	1	3	1	1	–	6	2.36	2	1	–	3	1.18	9	3.54 ± 1.41^a^	56.45** ± **5.43^ab^

*NC* negative control, *ATZ* atrazine, *PE* plant extract, *TC* total cells, *TDC* total dividing cells, *NC* normal cells, *Cm* C-mitosis, *St* stickiness, *Vg* vagrant, *Lg* laggard, *Da* delayed anaphase, *Cf* chromosome fragments, *Cb* chromosome bridge, *Ck* chromosome break, *TPA* total physiological aberration, *TCA* total clastogenic aberrations.

Data are mean ± SD of three replicates. Different alphabets (a and b) are significantly different at (p < 0.05), as determined by Tukey HSD test.

Furthermore, in the present study sodium azide was used as a positive control. Among all the tested concentrations (1, 5 and 10 µg/mL) of sodium azide, the maximum reduction in root growth and mitotic index, as well as increase in chromosomal aberrations, were observed at a concentration 5 µg/mL of sodium azide (Tables 3, 4 and 5).

The ameliorating activity of aqueous extract of leaves of *Roylea cinerea* (D.Don) Baillon against the effective concentration (10 µg/mL) of atrazine in the root tips cells of *Allium cepa* was also studied. For this, two modes of treatment: pre and post were given. In pre-treatment, the highest percentage of mitotic index value (54%) was found to be at highest concentrations of extract and atrazine. In post-treatment, the highest percentage of mitotic index value (53%) was also found to be at highest concentrations of atrazine and extract (Table 8). The data of total root growth also showed a great increase (Table 7). The maximum increase in total root growth was recorded as (72.64%) for pre-treatment and (76.33%) for post-treatment in comparison to (52.33%) for atrazine alone treatment. Moreover, a great reduction in the number of both physiological and clastogenic chromosomal aberrations was also observed when the root tips of *Allium cepa* were treated with the aqueous extract of leaves of *Roylea cinerea* (D.Don) Baillon (Table 8). Based on the observations, it was found that the extract of leaves of *Roylea cinerea* (D.Don) Baillon in both pre-and post-treatment was highly capable of reducing the percentage of chromosomal aberrations induced by atrazine.

**Table 7:** Combine effects of atrazine, sodium azide and plant extract on *Allium cepa* root growth.

Treatment groups	Concentrations µg/mL	Average root number ± SD	Average root length (cm) ± SD	TRG (%) ± SD	Percentage inhibition of TRG ± SD
NC	Tap water	47.67 ± 5.86^d^	5.07 ± 0.07^c^	100.00 ± 0.00^b^	0.00 ± 0.00^ab^
ATZ	10	19.33 ± 10.07^ab^	2.69 ± 0.36^a^	52.33 ± 6.91^a^	47.63 ± 6.88^b^
SA	5	18.33 ± 6.11^a^	3.21 ± 0.17^ab^	62.48 ± 3.40^a^	37.52 ± 3.40^b^
PE + ATZ (PRE)	0.25 + 10	32.67 ± 7.02ab^cd^	3.81 ± 0.22^b^	52.10 ± 36.00^a^	47.90 ± 36.00^b^
	0.5 + 10	39.33 ± 6.51^cd^	3.60 ± 0.27^ab^	69.99 ± 5.24^ab^	30.01 ± 5.24^ab^
	1.0 + 10	40.67 ± 3.51^cd^	3.52 ± 0.23^ab^	68.43 ± 4.51^ab^	31.57 ± 4.51^ab^
	1.5 + 10	38.00 ± 2.65^cd^	3.50 ± 0.37^ab^	68.12 ± 7.13^ab^	31.88 ± 7.13^ab^
	3.0 + 10	40.33 ± 2.52^cd^	3.73 ± 0.52^ab^	72.64 ± 10.15^ab^	27.36 ± 10.15^ab^
ATZ + PE (POST)	10 + 0.25	29.67 ± 4.16^abc^	3.92 ± 0.21^b^	76.33 ± 4.00^ab^	23.67 ± 4.00^ab^
	10 + 0.5	34.67 ± 6.66^bcd^	3.17 ± 0.21^ab^	61.67 ± 4.02^a^	38.33 ± 4.02^b^
	10 + 1.0	38.00 ± 3.61^cd^	2.95 ± 0.44^ab^	57.46 ± 8.49^a^	42.54 ± 8.49^b^
	10 + 1.5	31.33 ± 4.04^abc^	3.08 ± 0.49^ab^	59.92 ± 9.44^a^	40.08 ± 9.44^b^
	10 + 3.0	39.00 ± 2.00^cd^	3.49 ± 0.67^ab^	67.96 ± 13.05^ab^	32.04 ± 13.05^ab^

*NC* negative control, *ATZ* atrazine, *SA* sodium azide, *PE* plant extract, *TRG* total root growth. Data are mean ± SD of three replicates. Different alphabets (a, b, c and d) are significantly different at (p < 0.05), as determined by Tukey HSD test.

**Table 8:** Antigenotoxic potential of aqueous extract of leaves of *Royale cinerea* (D.Don) Baillon by *Allium cepa* root tip assay with pre- and post-treatment against the genotoxicity induced by atrazine.

Sample code Conc. µg/mL		TC	TDC	NC	Physiological aberrations (PA)							Clastogenic aberrations (CA)							MI ± SD
					Cm	St	Vg	Lg	Da	TPA		Cf	Cb	Ck	TCA		TPA ± TCA		
										No.	%				No.	%	No.	%	
NC	Tap water	450	271	264	2	2	–	1		5	1.85	1	–	1	2	0.74	7	2.59 ± 0.56^a^	60.22 ± 7.19^a^
ATZ	10	450	143	54	24	16	7	4	5	56	39.16	11	17	5	33	23.08	89	66.01 ± 24.49^a^	31.78 ± 8.34^a^
PRE	0.25 + 10	450	154	143	1	2	1	1	1	6	3.90	2	1	2	5	3.25	11	7.38 ± 5.77^a^	34.22 ± 16.39^ab^
	0.5 + 10	450	173	159	1	2	3	1	2	9	5.20	1	2	2	5	2.89	14	8.32 ± 4.47^a^	38.44 ± 4.44^ab^
	1.0 + 10	450	147	137	1	1	–	1	–	3	2.04	3	2	2	7	4.76	10	8.24 ± 6.83^a^	32.67 ± 12.72^ab^
	1.5 + 10	450	215	205	2	1	1	1	–	5	2.33	3	1	1	5	2.33	10	5.51 ± 3.49^a^	47.78 ± 18.04^ab^
	3.0 + 10	450	243	236	1	1	1	1	1	5	2.06	–	1	1	2	0.82	7	3.23 ± 1.84^a^	54 ± 12.22^ab^
POST	10 + 0.25	450	152	139	2	1	1	1	1	6	3.95	2	3	2	7	4.61	13	9.12 ± 3.27^a^	33.78 ± 9.58^ab^
	10 + 0.5	450	169	141	1	2	1	2	1	7	4.14	2	1	1	4	2.37	11	11.29 ± 14.56^a^	37.56 ± 18.68^ab^
	10 + 1.0	450	155	137	3	1	1	2	2	9	5.81	2	2	2	6	3.87	15	11.89 ± 6.23^a^	34.44 ± 19.16^ab^
	10 + 1.5	450	219	140	1	1	2	2	–	6	2.74	3	1	2	6	2.74	12	6.78 ± 6.00^a^	48.67 ± 17.68^ab^
	10 + 3.0	450	240	143	1	1	1	1	–	4	1.67	1	1	3	5	2.08	9	3.91 ± 2.63^b^	53.33 ± 5.70^b^

*NC* negative control, *ATZ* atrazine, *SA* sodium azide, *PRE* pretreatment, *Post* posttreatment, *TC* total cells, *TDC* total dividing cells, *NC* normal cells, *Cm* C-mitosis, *St* stickiness, *Vg* vagrant, *Lg* laggard, *Da* delayed anaphase, *Cf* chromosome fragments, *Cb* chromosome bridge, *Ck* chromosome break, *TPA* total physiological aberration, *TCA* total clastogenic aberrations.

Data are mean ± SD of three replicates. Different alphabets (a and b) are significantly different at (p < 0.05), as determined by Tukey HSD test.

**Figure 3:**
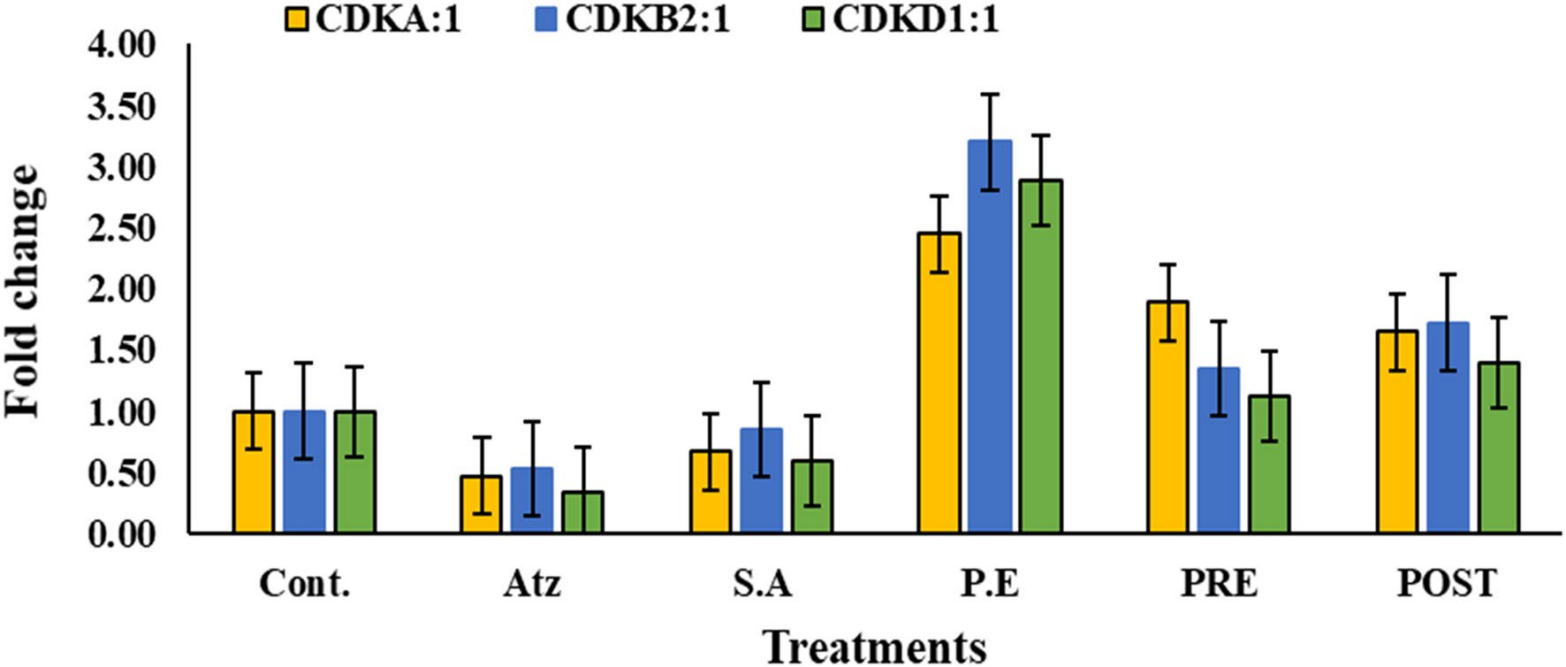
Effects of treatments with atrazine (ATZ), sodium azide (S.A), plant extract (P.E), Pre-treatment (PRE) and Post-treatment (POST) on CDKA:1, CDKB:2, CDKD1:1 fold change expression level in the root tip cells of *Allium cepa*. All values are relative to average expression level, of the root tips cells of *Allium cepa* treated with the tap water, atrazine, sodium azide, PE, PRE and POST treatment in triplicate, n = 3 and mean ± SE.

To understand the mechanism of genoprotective activity of aqueous extract of leaves of *Roylea cinerea* (D.Don) Baillon in reducing the genotoxic effect of atrazine, RT-qPCR studies were conducted. For this, RNA was extracted from the treated root tips and subjected to RT-qPCR analysis. It was observed that the expression of marker genes; Cyclin-dependent kinases (CDKA:1, CDKB2:1, and CDKD1:1) for the atrazine-treated roots was found to be lowered in comparison to control. Whereas the expression level was found to be upregulated on treatment with the aqueous extract of leaves of *Roylea cinerea* (D.Don) Baillon (Fig. 3). The mean values of expression levels in folds change for CDKA:1, CDKB2:1 and CDKD1:1 marker genes was found to be 1.0, 0.47, 0.67, 2.45, 1.89 and 1.65 for (CDKA:1), 1.0, 0.53,0.85,3.20,1.35 and 1.72 for (CDKB2:1) and 1.0, 0.34,0.60,2.89, 1.12 and 1.40for (CDKD1:1) for control, atrazine, sodium azide, plant extract alone and pre and post-treatment with aqueous extract of leaves of *Roylea cinerea* (D.Don) Baillon respectively.

## Discussion

The genotoxicity of atrazine and antigenotoxicity of aqueous extract of leaves of *Roylea cinerea* (D.Don) Baillon were tested on the root tip cells of *Allium cepa.* The *Allium* test has been widely used by many investigators to study the biological monitoring, environmental pollution investigation, genotoxicity, and antigenotoxicity potential of many medicinal plants^38–42^.

Results obtained from the effect of atrazine on the root growth of *Allium cepa* and mitotic index value showed that there was a concentration-dependent decrease in root growth and mitotic index values as compared to control. The genotoxic effect of the different concentrations of atrazine on the mitotic cell division of the root tip cells of *Allium cepa* is given in Table 3. It was found that an increase in atrazine concentration resulted in a corresponding decrease in mitotic index. After 24 h duration treatment, the mitotic index was found to be lowest at 10 µg/mL concentration of atrazine in comparison to control. Moreover, the percentage frequency of aberrant cells was also found to be highest at tested concentrations (10 µg/mL) for atrazine. This significant (p ≤ 0.05) reduction with increasing concentration of atrazine suggests that the tested chemical can cause cytotoxic effects in *Allium cepa* and, for all exposed organisms to such herbicide. Moreover, it was also observed that the higher the concentration used, the maximum were the chromosomal aberrations in the root tip cells treated with atrazine. Our results are in agreement with the study conducted by others investigators on the genotoxic nature of atrazine on the root tip cells of *Allium cepa*^1,6,43–46^.

The largest percentage of abnormal cells was found at a dosage of 10 µg/mL of atrazine used, indicating that higher concentrations of this herbicide are more genotoxic, caused the maximum chromosomal abnormalities and can thus be considered as the most effective concentration for atrazine genotoxicity.

The different types of chromosomal aberrations induced by atrazine herbicide in root tip cells of *Allium cepa* are given in Fig. 2. In the genotoxic investigation, root tip cells treated with atrazine showed a significant rise in both physiological and clastogenic aberration with the increase in tested concentrations. The types of abnormalities were seen in different mitotic stages in all treated root tips include; c-metaphase, stickiness at telophase, laggard chromosomes, vagrant chromosomes, chromatin bridge, chromosomes fragments/breaks, and delayed anaphase with bridge Similarly, numerous researchers observed these types of chromosomal aberrations after treating *Allium cepa* with various S-triazine herbicides^47–51^.

In the present study, c-metaphase and chromosome stickiness were the two major chromosomal aberrations recorded. Both these types may be formed due to the herbicide's (atrazine) influence on the polymerization and spiralization processes. This sticky chromosome leads to the formation of chromosome fragmentation and bridge at the ana-telophase stage.

Another sign of atrazine's genotoxicity is the c-metaphases. The nuclear chromosome is entirely inactivated during c-metaphases or c-mitosis which means that no equatorial plate forms and as a result, centromere division is slowed or even prevented. C-metaphase or c-mitosis also leads to a large divergence of the chromosome number causing chromosome imbalance, chromosome loss, partial or complete inactivation of the chromosome spindle, and accelerated chromosome contraction. Thus c-metaphase should be considered as a major harm mechanism and included in the parameters used to screen for genotoxic and mutagenic chemicals^34,52^.

Chromosome laggards, anaphase, and telophase bridges were also observed. Anaphase bridges can be caused by translocations or merely from cohesive chromosomal ends. Bridges can also be formed as a result of chromosome adhesions, and they can be numerous and last until telophase. The chromosome bridges are formed as a result of structural rearrangements and chromosome breakage^53^.

Our results of chromosomal aberrations are in agreement with other toxicological studies that have shown the ability of atrazine to induce genotoxic/mutagenic effects in a variety of organisms^6,54–58^.

Several medicinal plants and their products are being used today to ameliorate the toxic effects caused by different toxicants. Anti-mutagenic, anti-genotoxic, and anticarcinogenic activities have been described for plants and their products^59,60^. Antigenotoxicity of an antigenotoxic agent may be defined as its ability to minimize genotoxicity in an organism caused by genotoxicants.

In the present study, the antigenotoxic activity of aqueous extract of leaves of *Roylea cinerea* (D.Don) Baillon alone in the root tips cells of *Allium cepa* was also determined. Our data showed that all the different concentrations of extract (0.25, 0.5, 1.0, 1.5, and 3.0 µg/mL) alone in comparison to atrazine (10 µg/mL) concentration caused an increase in root growth and mitotic index. It was also found that with the increase in the extract concentrations, the number of chromosomal aberrations also decreased showing the antigenotoxic potential of extract (Table 6). The results of pre and post-treatments are given in Table 7. It was found that the average root number, average root length, and total root growth percentage increased with both pre and post-treatment in comparison to the atrazine alone treatment. The results of the percentage frequency of aberrant cells are given in Table 8 which indicates that the percentage frequency of chromosomal aberrations decreased with the pre and post-treatment in comparison to the atrazine treatment, but a higher reduction was found more in the pre-treatment. Moreover, the mitotic index value also showed an increase with pre and post-treatment in comparison to atrazine alone but higher mitotic index values were seen with the higher concentrations of the aqueous extract of leaves of *Roylea cinerea* (D.Don) Baillon used.

As the results of the present study showed that all the tested concentrations of the aqueous extract of leaves of *Roylea cinerea* (D.Don) Baillon alone as well as in both the treatments viz. pre and post reduce the number of aberrant cells in comparison to the atrazine. This inhibitory potential of the extract may be due to the different phytoconstituents present. Many phytochemicals such as polyphenols, tannins, flavonols, and alkaloids, all naturally occurring chemicals in plants, have been shown to reduce chromosomal damage at higher doses. Literature findings on the antimitotic action of *Rhodiola rosea* L. roots extracts using the *Allium cepa* test, supported the results of present findings^38^. This is also in consistent with several prior publications on the phytochemical examination of the plant extracts studied. Some researchers conducted similar studies and discovered that plant extracts had an antigenotoxic effect against the toxicity of certain toxicants or mutagens^61–67^.

Furthermore, in the present study, total phenolic content (TPC) for aqueous extract of leaves of *Roylea cinerea* was found to be 113.36 µg GAE/mg dry weight of the extract and the total flavonoid content (TFC) was obtained as 140.44 µg RE/mg dry weight of the extract respectively. These values of total phenolic content (TPC) and the total flavonoid content (TFC) indicate that the aqueous extract of leaves of *Roylea cinerea* is highly rich in phenolic and flavonoid compounds. So, the antigenotoxic potential of the leaves extract may be due to the presence of these phenolic and flavonoid compounds. A review of the literature revealed that many medicinal plants with high phenolic content have been linked to chemopreventive and anticancer activity^68–72^.

The preliminary phytochemical analysis of the aqueous extract of leaves of *Roylea cinerea* with high-performance liquid chromatography (HPLC) showed the presence of eight polyphenol compounds viz; Gallic acid, Chlorogenic acid Epicatechin, Caffeic acid, Umbelliferone, Coumaric acid, tert-Butyl hydroquinone and Kaempferol (Fig. 1). This may be another reason for reducing the chromosomal aberrations caused by the herbicide atrazine. A similar study was also conducted where seven polyphenols including gallic acid, rutin, catechin, quercetin, umbelliferone, epicatechin, and kaempferol were investigated from the stem and leaves extract of *Roylea cinerea*^73^*.* The anti-oxidant and antiproliferative activities of *Roylea cinerea* were also conducted, where the methanolic extracts of leaves and stem of *Roylea cinerea* were used for the investigation of antioxidant activity using hydrogen/electron-donating and hydroxyl radical scavenging assay and antiproliferative and apoptotic activity on L6 rat skeletal muscle cell line^33^. The in vitro studies showed that the bioactive compounds present in the methanolic extracts of leaves of *Roylea cinerea* possess anticancer potential.

Furthermore, the study was also carried out to examine the mechanism of genoprotective activity of aqueous extract of leaves of *Roylea cinerea* (D.Don) Baillon in reducing the genotoxic effect of atrazine by analyzing the expression of marker genes i.e. Cyclin-dependent kinase (CDKs) (CDKA:1, CDKB2:1, and CDKD1:1) from the root tip cells of *Allium cepa* by using RT-qPCR. These Cyclin-dependent kinases (CDKs) are known to be the major regulators of the eukaryotic cell cycle. In response to phytohormonal signals, they are thought to influence cell differentiation and proliferation^74^.

CDKA:1, CDKB2:1, and CDKD1:1 were chosen because they play a significant role in the regulation of the cell cycle at the G1/S and G2/M transitions. CDKD was chosen because it is part of a kinase network that controls CDK activity through a phosphorylation cascade. It phosphorylates a conserved threonine residue in the T-loop region to activate another plant's CDKs^75^. The significant reduction in the mitotic index in pesticide-treated *Allium cepa* root tip cells indicated probable CDKs inhibition, as previously observed due to the presence of cyclin-dependent kinase inhibitors in many fungicides and insecticides^76^.

In the present study, the expression levels of CDKA:1, CDKB2:1, and CDKD1:1 for the atrazine-treated roots decreased in comparison to control (Fig. 2). More reduction was found in the expression level of CDKB2:1 among all the targeted genes. The CDK1 complex is required for cell progression during the M phase and is involved in the creation of the mitotic spindle. Reduced expression of CDKA:1, CDKA2:1, and CDKD1:1 reduces cell division in the M phase and causes various chromosomal abnormalities owing to spindle formation disruption. CDK2, or cell division protein kinase 2, on the other hand, is known to be involved in the G1/S transition as well as the progression through the S phase. Cell cycle arrest and cell division are slowed when CDK2 protein levels are low^77^.

These findings were also in accordance with the mitotic cell division studies, which showed that induction of chromosomal abnormalities, as well as reduction in mitotic index (Table 3) in *Allium cepa* root tip cells samples from pesticide-treated (atrazine), can be linked to lower levels of CDKA:1, CDKB2:1 and CDKD1:1genes under similar conditions. Our results of the study are in agreement with the previous studies done where pesticide treatment reduces the expression levels of targeted genes^46,77,78^.

It was also noted that the aqueous extract of leaves of *Roylea cinerea* alone increases the expression level of CDKs in comparison to atrazine treatments (Fig. 2). Also, the expression levels of the target genes were found to be higher in pre and post-treatment in comparison to atrazine-treated root tip cells of *Allium cepa.* Our results of the study are in agreement with the study conducted by other authors in which, in comparison to atrazine treatments, treatment with N–Se resulted in a significant increase in the expression level of all examined genes^46^. It can be inferred that using an aqueous extract of *Roylea cinerea* leaves in both the pre and post-treatment lowers the genotoxic effect of atrazine on all of the genes tested.

## Conclusion

To conclude, the present study indicates the antigenotoxic potential of aqueous extract of leaves of *Roylea cinerea* (D.Don) Baillon against the genotoxicity of atrazine on the root tip cells of *Allium cepa*. Phytochemical analysis has shown the presence of various active ingredients in this plant. To the best of our knowledge, this study is the first of its kind to bring out the antigenotoxic potential of the aqueous extract of leaves of *Roylea cinerea* (D.Don) Baillon in the plant system. This antigenotoxic feature of the extract is may be due to the presence of eight polyphenolic compounds viz; Gallic acid, Chlorogenic acid Epicatechin, Caffeic acid, Umbelliferone, Coumaric acid, tert-Butyl hydroquinone, and Kaempferol. Furthermore, this research reveals that an aqueous extract of *Roylea cinerea* (D.Don) Baillon leaves could be used to create anticarcinogenic drugs. Additional antigenotoxicological research is required for the welfare of human well-beings.

## Supplementary Information

Supplementary Table S1.

## Data Availability

The raw data supporting the conclusions of this article will be made available by the corresponding author, without undue reservation, to any qualified researcher.

## Abbreviations

CA: Clastogenic aberrations
CDKs: Cyclin-dependent kinases
ESTs: Expressed sequence tags
HPLC: High-performance liquid chromatography
NCBI: National Center for Biotechnology Information
PA: Physiological aberrations
RT-qPCR: Real time-quantitative polymerase chain reaction
