# Supplementary material for: Modulation of atrazine-induced chromosomal aberrations and cyclin-dependent kinases by aqueous extract of Roylea cinerea (D.Don) Baillon leaves in Allium cepa
Source: Sci Rep. 2022 Jul 22;12:12570. doi: 10.1038/s41598-022-16813-z (PMC9307653; doi:10.1038/s41598-022-16813-z)
Supplement: Supplementary file 1 — Supplementary Table S1. [file 41598_2022_16813_MOESM1_ESM.pdf]

Modulation of atrazine-induced chromosomal aberrations and cyclin-dependent kinases by Aqueous extract of *Roylea cinerea* (D.Don) Baillon leaves in *Allium cepa*

Farhana Rashid[1], Davinder Singh[1], Shivani Attri[1], Prabhjot Kaur[1], Harneetpal Kaur[1], Pallvi Mohana[1], Jahangeer Quadar[1], Adarsh Pal Vig[1], Astha Bhatia[1], Balbir Singh[2], Harpreet Walia[3], Saroj Arora[1*]

[1] *Department of Botanical and Environmental Sciences, Guru Nanak Dev University, Amritsar, Punjab (India)*

[2] *Department of Pharmaceutical Sciences, Guru Nanak Dev University, Amritsar, Punjab (India)*

[3] *Department of Botany and Environment Studies, DAV University, Jalandhar, Punjab (India)*

***Corresponding Author**

Dr. Saroj Arora, Professor,

Department of Botanical & Environmental Sciences,

Guru Nanak Dev University,

Amritsar-143005, Punjab (India)

Email: dr.sarojarora@gmail.com; sarojarora.gndu@gmail.com

Contact: +91-9877618922

Table. S1 . Polyphenols concentrations detected in aqueous extract of leaves of *Royale cinerea* (D.Don) Baillon

Peak Table

PDA Ch1 280nm

Peak#	Ret. Time	Area	Height	Conc.	Unit	Mark	Name
1	1.620	2354061	178396	0.000			
2	2.089	399446	47692	0.000		V	
3	2.264	1981829	365905	0.000		V	
4	2.627	1365656	57942	95.775	mg/L	V	Gallic acid
5	3.230	105582	11826	0.000		V	
6	3.469	407671	13823	0.000		V	
7	4.656	3677	539	0.481	mg/L	V	Chlorogenic acid
8	5.087	7128	553	0.000			
9	6.365	5453	481	1.433	mg/L		Epicatechin
10	6.622	4375	271	0.314	mg/L		Caffeic acid
11	9.531	104298	2334	22.294	mg/L		Umbelliferone
12	10.376	5899	164	0.201	mg/L	V	Coumaric acid
13	10.926	1216	120	0.000			
14	16.149	2737992	27029	437.784	mg/L		tert-Butyl hydroquinone
15	17.272	1053840	20264	449.027	mg/L	V	Kaempferol
16	21.304	193078	4658	0.000			
17	21.352	28939	4652	0.000		V	
18	21.463	148085	4638	0.000		V	
19	22.480	134808	3980	0.000		V	
20	22.560	188268	3914	0.000		V	
Total		11231302	749182				

Gallic acid: 95.775 mg/L; Chlorogenic acid: 0.481 mg/L; Epicatechin: 1.433 mg/L; Caffeic acid:0.314; Umbelliferone: 22.294 mg/L; Coumaric acid:0.201 mg/L; **tert-Butyl hydroquinone: 437.784 mg/L**; **Kaempferol: 449.027 mg/L.**
